# Clinical implementation of dynamic intensity-modulated radiotherapy: Dosimetric aspects and initial experience

**DOI:** 10.4103/0971-6203.41195

**Published:** 2008

**Authors:** S. S. Sivakumar, K. Krishnamurthy, C. A. Davis, R. Ravichandran, S. Kannadhasan, J. P. Biunkumar, Kamal El Ghamrawy

**Affiliations:** Department of Radiation Oncology, National Oncology Centre, the Royal Hospital, Muscat, Sultanate of Oman

**Keywords:** Dose verification, intensity-modulated radiotherapy, millennium multi-leaf collimator, quality assurance test, sliding window

## Abstract

This paper describes the initial experience of quality assurance (QA) tests performed on the millennium multi-leaf collimator (mMLC) for clinical implementation of intensity-modulated radiotherapy (IMRT) using sliding window technique. The various QA tests verified the mechanical and dosimetric stability of the mMLC of linear accelerator when operated in dynamic mode (dMLC). The mechanical QA tests also verified the positional accuracy and kinetic properties of the dMLC. The stability of dMLC was analyzed qualitatively and quantitatively using radiographic film and Omnipro IMRT software. The output stability, variation in output for different sweeping gap widths, and dosimetric leaf separation were measured. Dose delivery with IMRT was verified against the dose computed by the treatment planning system (TPS). Monitor units (MUs) calculated by the planning system for the IMRT were cross-checked with independent commercial dose management software. Visual inspection and qualitative analysis showed that the leaf positioning accuracy was well within the acceptable limits. Dosimetric QA tests confirmed the dosimetric stability of the mMLC in dynamic mode. The verification of MUs using commercial software confirmed the reliability of the IMRT planning system for dose computation. The dosimetric measurements validated the fractional dose delivery.

## Introduction

Development of digital linear accelerators with multi-leaf collimator and inverse planning algorithms has given a new dimension to radiation dose delivery. Integration of these two latest technologies helped to optimize the dose delivery to target volume, while sparing organs at risk (OAR) by a novel technique known as intensity-modulated radiotherapy (IMRT).[1] IMRT is increasingly becoming the standard of care in cancer treatment even in developing countries.

Dose delivery by IMRT requires operation of multi-leaf collimators (MLC) either in step-and-shoot mode or dynamic dose mode. In step-and-shoot mode, the shaped MLC remains fixed while the beam is on, and modulation of the beam is achieved through a series of complex small-segmented subfields. In dynamic dose mode, each leaf pair of MLC moves continuously, unidirectionally, and with independent speed while the beam is on, to modulate the beam. The flux in the individual subfields remains constant in the former mode in contrast to variable flux in pencil beams for the latter during sliding of vanes.

Dosimetric precision of IMRT in dynamic mode is primarily dependent on the positional accuracy of each leaf during the entire treatment period. The dose distribution is computed by inverse planning in the treatment planning system (TPS), and delivery sequence is exported to the linear accelerator. Importance of testing the MLCs and understanding their physical and dosimetric aspects has been reported in literature.[2,3] In developing countries, IMRT is not practiced generously due to technological limitations, and many physicists are not very familiar with IMRT planning software.

Independent validation of calculation done by the TPS is recommended.[4] The associated hardware and software components of IMRT system need to be rigorously checked, and QA tests have to be planned, prior to its clinical implementation. Keeping in view the fact that the accuracy of dose delivery in IMRT depends on the mechanical and dosimetric stability of MLCs, extensive QA tests need to be preformed.

Our experience in commissioning of IMRT and QA tests for clinical implementation using sliding window technique is presented.

## Materials and Methods

Clinac 2300 CD (M/s Varian AG, USA) with millennium 120 MLC as tertiary collimator was used for sliding window IMRT technique. The outer pairs of 20 leaves have a thickness of 1 cm, and the inner pairs of 40 leaves have a thickness of 5 mm, with provision for variable speed.

For IMRT, 6-MV photon with beam quality ( Tissue Phantom Ratio – (TPR _20/10_ – 0.6718) was used.

Relative dosimetry was performed using Kodak X Omat V ready-pack film (Eastman Kodak Company, USA), VXR16 film scanner (VIDAR Systems, USA), and Omnipro IMRT software (Scanditronix Wellhofer, Germany). Calibration of films was carried out by exposing the films to doses ranging from 25 to 300 cGy. Development of films was done in automatic film processor (Agfa Gevaert, Germany). For absolute dosimetry, solid water phantom and Dose1 electrometer with Farmer ion chamber (FC65 — nominal volume, 0.65 cc; and total active length, 2.3 cm) were used. IMRT planning and dose computation were performed by Eclipse treatment planning system (M/s Varian AG, USA) with inverse planning Helios optimization software. The treatment plan was exported to ‘Diamond’ (K and S Associates Inc., USA) dose calculation management software. ‘Diamond’ software can independently compute dose and MUs at reference points. These parameters for standard dMLC test pattern plans calculated by the Eclipse TPS were cross-checked using ‘Diamond.’

### A. Mechanical quality assurance tests

The mechanical and dosimetric quality assurance tests done during commissioning are listed in Table 1. The methodology and purpose of each of these standardized tests are well documented and available in literature.[5,6] The functional accuracy of leaf pairs and their kinetic properties are verified by standard dMLC test patterns.[7] The test patterns are intended to give specific density pattern on radiographic film when exposed. The objectives of these tests are summarized in Table 2, column 3.

**Table 1:** Mechanical and dosimetric quality assurance tests

A. Mechanical Quality Assurance Tests
1. Stability of dMLC
a) Picket Fence Test
b) Garden Fence Test
2. Leaf speed and Stability test (with and without beam interruption)
3. Evaluation of standard patterns
a) Synchronized and Non Synchronized Segmented Stripes
b) X wedge
c) Y Wedge
d) Pyramids
e) Complex fields
4. Additional test patterns (Chess shape, “h” letter shape)
B. Dosimetric Quality Assurance Tests
1. Output Stability
2. Variation in output for Different Sweeping Gap width
3. Determination of Dosimetric Leaf Separation
4. Dosimetric verification of the IMRT Delivery System
5. Validation of IMRT Treatment Plans

**Table 2:** Mechanical quality assurance tests

*SI. No*	*Name of the DMLC QA pattern test*	*Objective*	*Acceptable specification*	*Matchline*
			
			*Manufactured quoted*	*Observed on measurement*
1	Picket Fence	Verifies leaf and carriage position accuracy and calibration	Matchline at every 5 ± 0.1 cm	at every 5 ± 0.1 cm
2	Garden Fence	Verifies leaf and carriage position accuracy and calibration	Matchline at every 2 ± 0.1 cm	at every 2 ± 0.1 cm
3	Synchronized Segmented Strip	Verifies the accuracy and calibration of the leaf position and carriage movement, when some adjacent leaf pairs are closed during beam delivery, effects of inter-leaf friction on leaf positioning and the ability of the leaves to interdigitate	Match line at every 4 ± 0.1 cm	at every 4 ± 0.1 cm
4	Non- synchronized Segmented Strip	Verifies the leaf position accuracy and calibration and detect possible effects of interleaf friction in case of non-synchronized leaf motion	Matchline at every 2 ± 0.1 cm	at every 2 ± 0.1 cm
5	X Wedge	Verifies the leaf speed stability, acceleration and deceleration	Matchline at every 2 ± 0.1 cm	at every 2 ± 0.1 cm
6	Y Wedge	Verifies the leaf speed stability, acceleration and deceleration	Matchline at every 2 ± 0.1 cm	at every 2 ± 0.1 cm
7	Pyramid shape	Verifies the accuracy and calibration of the leaves in producing complex pyramid fields.	Matchline at every 1 ± 0.1 cm	at every 1 ± 0.1 cm
8	Complex fields	Verifies the ability of dMLC to produce complex intensity modulated pattern	Matchline between different intensity segment should be straight	Field boundaries are well defined and matchline is straight

These test patterns were evaluated qualitatively by visual inspection and by transferring them to Omnipro IMRT software. Complex field shapes (chess pattern, letter ‘h’ shape) were created using standard text editor, and the dose distribution was calculated by the TPS. Correctness of leaves during sliding movements was documented by DynaLog file viewer software[8] available in control console computer. Accuracy of leaf position was periodically verified with electronic portal imaging device (EPID).

### B. Dosimetric quality assurance tests

1. Output stability: To verify the output stability of the linac under dynamic mode, a dMLC test file of fixed gap width (1 cm) was created with shaper software and swept over 10 cm. FC65 ion chamber with buildup material (wax cylinder of 15mm thickness, adequate for 6 MV X ray beam) was placed isocentrically at 100 cm in air. The chamber was exposed for 300 MU with sweeping field at a dose rate of 300 MU/min. The dosimeter reading was obtained for the center of the field and at off-axis distances of ±2.5 cm. The measurements were normalized to 10×10 static field with the same number of MUs (i.e., 300 MU). To evaluate the output stability under the influence of gravity on the leaves, the measurements were repeated for three different gantry angles (0°, 90°, and 270°).

2. Variation in output for different sweeping gap width: To determine the variations in output for different sweeping gap widths, dMLC files of 1.0 to 1.1 cm gap widths at 0.02 cm intervals were created and run. The dosimeter output was normalized to 1.0 cm sweeping field. A graph was plotted for sweeping gap width and relative output variation.

3. Determination of dosimetric leaf separation (DLS): The DLS was measured by ‘charite’ sweeping gap technique.[9] Five different dMLC files, with fixed leaf gap widths (0.5, 1, 4, 10, and 20 mm), sweeping at constant velocity to a total field size of 10×10 cm were created. FC65 ionization chamber with 15 mm wax buildup was positioned isocentrically in air at 100 cm. A graph was plotted for dynamic field gap vs. ion chamber readings. The DLS was obtained as the gap width intercept in abscissa. This value was configured in the TPS.

4. Dosimetric verification of IMRT delivery and computing system: Accuracy of the dose delivered by linac with dMLC was verified against dose planned by TPS. A solid water phantom cube (25×25×25 cm) was scanned with x-ray CT, and image was imported into the TPS. Dose distribution was calculated for dMLC test field for 100 cGy dose delivery at 5 cm depth. The treatment plan was exported to treatment machine through Varis-Vision network, and dose was delivered to a verification film kept at a depth of 5 cm in solid phantom. Dose distributions recorded by the film were compared with those calculated by TPS.

5. Validation of IMRT treatment plans: With DICOM RT plan export feature of Eclipse, dMLC test files and IMRT plans of the patient calculated by the TPS were transferred to the ‘Diamond’ software. Monitor units, fluence, and dose at reference point calculated by the DIAMOND software were compared with TPS-calculated dose parameters.

## Results

### A. Mechanical quality assurance tests

**Figure 1:**
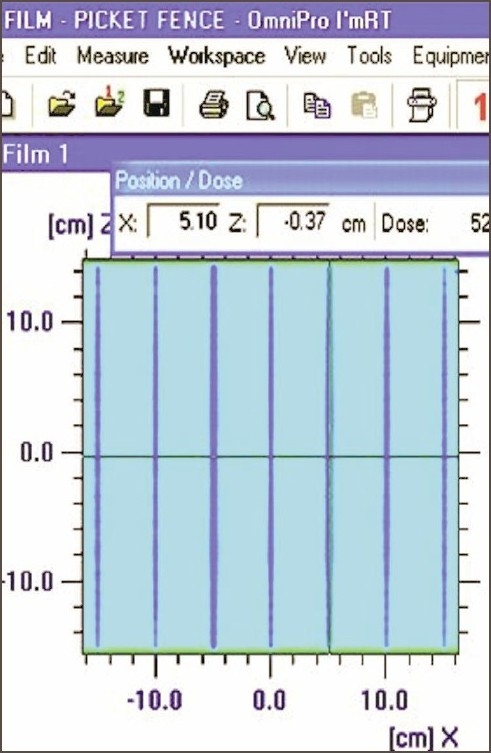
Match line separation appearance in the Picket Fence test

1. Movement of MLC during radiation beam exposure: The results observed for manufacturer-specified and dMLC test intensity patterns for their positional accuracy of match line are listed in Table 2. The qualitative analysis of standard dMLC patterns shows that the match lines between different intensity segments are straight, approximately equal in intensity, and lying within the positional error of ±0.1 cm. This implies that there is no leaf and carriage positional error occurring during the MLC movement. Match-line accuracy in Picket and Garden Fence test patterns also confirms the same. Figure 1 shows Picket Fence match line separated equally by 5±0.1 cm.

**Figure 2:**
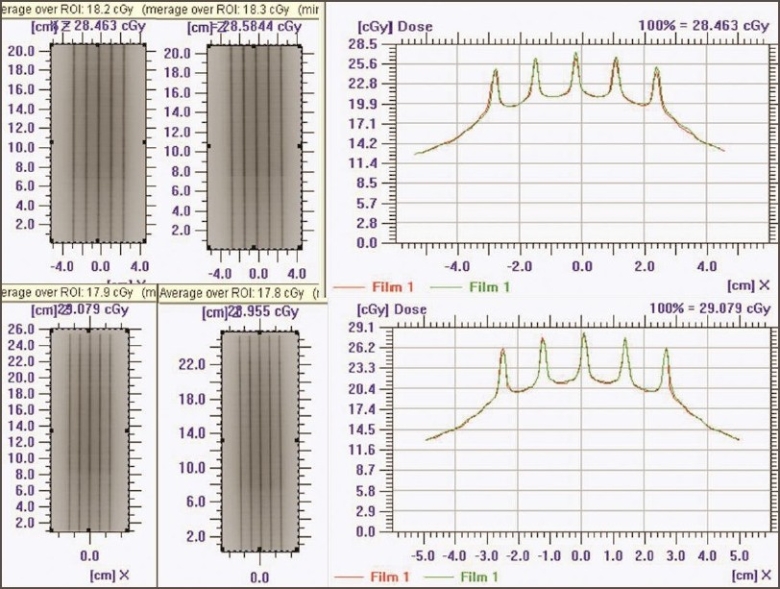
Recorded intensity pattern and superposition of dose profiles of Garden Fence test

**Figure 3:**
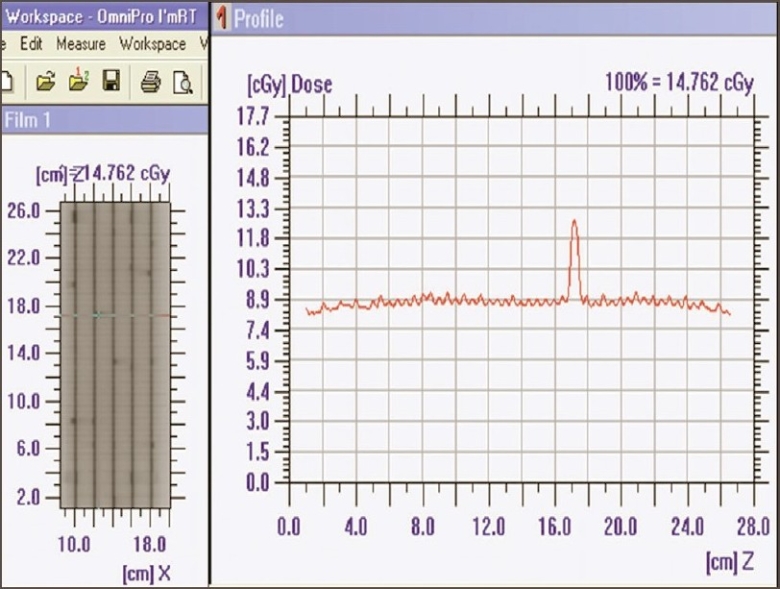
Intensity pattern and dose profile (perpendicular to direction of leaf movement) of Garden Fence test with errors in leaf positioning

The superposition of dose profile (along the direction of leaf movement) for Garden Fence test at 0°, 90°, 180°, and 270° gantry angles is seen in Figure 2. Leaf error histogram analysis using DynaLog File Viewer software, for the Garden Fence tests at 0°, 90°, 180°, and 270° gantry angles showed above 98% of error counts having misplacement <0.05 cm and no error count with misplacement >0.05 cm. This analysis also confirmed the mechanical stability of dMLC. Intentionally introduced errors in the Garden fence test were detectable by the film, even for the smallest leaf position misplacement, of the order of 0.05 cm. Figure 3 shows the intensity pattern for Garden Fence test (with intentional errors) and dose profile. EPID was found to provide verification of leaf position accuracy.

**Figure 4:**
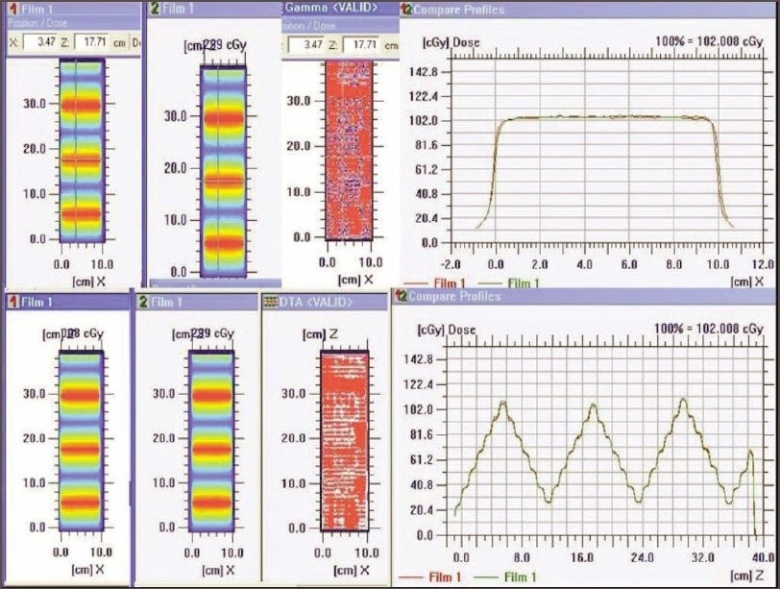
Intensity pattern of leaf speed and stability test (with and without beam interruption (left top and bottom). Superposition of dose profile (parallel and perpendicular to the direction of leaf movement) with and without beam interruption (film 1 without beam interruption, film 2 with beam interruption)

**Figure 5:**
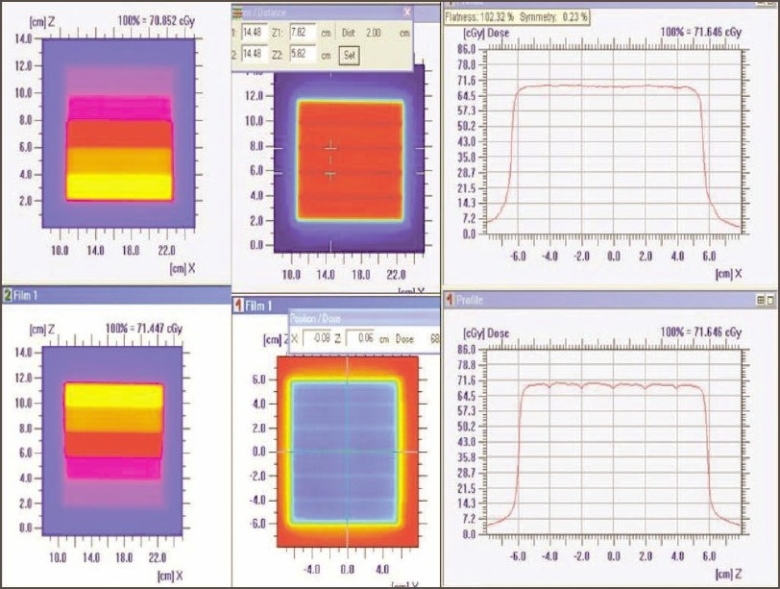
Complementary varying density pattern for Y and inverted Y wedge field (top and bottom left). Match line separation at 2 cm shown with Y and inverted Y wedge field irradiated on a single film (top and bottom middle). Dose profile along the match line (top right). Dose profile perpendicular to the match line (bottom right)

**Figure 6:**
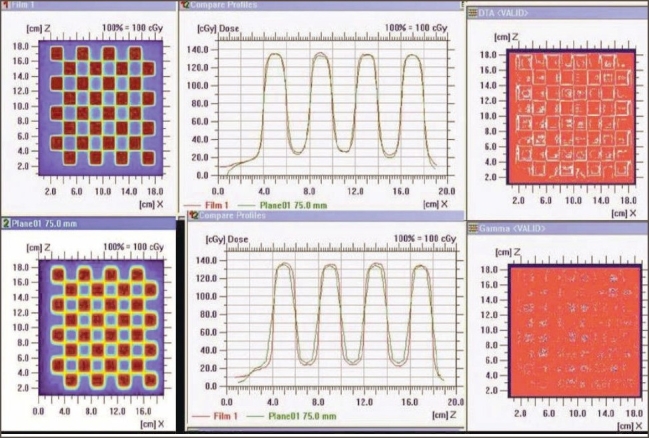
Intensity pattern of chess shape (top left — film; bottom left — TPS). Superimposed dose profile (parallel to the direction of leaf movement and perpendicular to the direction of leaf movement)

2. Leaf speed and stability test: Qualitative analysis of dose profiles for leaf speed and stability test with and without beam interruptions showed that they were identical and well within the uncertainty of film dosimetry. This is demonstrated in Figure 4, showing the superimposition of dose profiles parallel and perpendicular to the leaf motions. Thus it was observed that leaf speed remained constant and dose delivered was not affected by the interruptions.

3. Standard dMLC delivery patterns: Figure 5 shows Y and inverted Y wedge patterns recorded on a single film. The match line was seen separated equally at 2±0.1 cm (on either side from the center of the field). The match line coincided with the lines of interleaf leakage. Also the varying intensity patterns of Y and inverted Y wedge fields complemented each other. Figure 6 shows the comparison of dose profile (along the direction of leaf) of chess pattern calculated by TPS with that recorded in film. The dose profile (along the direction of leaf) measured on the film was found comparable with the TPS calculation, confirming positioning accuracy of leaves and carriage for large field size, when the carriage movement occurs.

**Figure 7:**
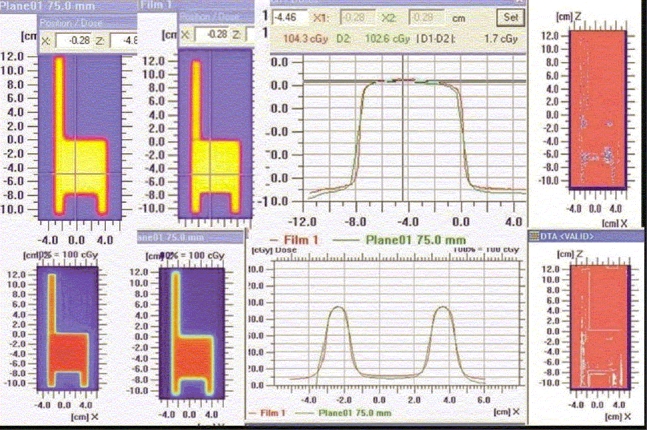
‘h’-shaped density pattern (top left — film; top middle — TPS) Superimposed dose profile — parallel to the direction of leaf movement, in the uniform dose region of chair (top right); superimposed dose profile — parallel to the direction of leaf movement, in the leg region of chair (bottom left); and quantitative evaluation of superimposed dose profile with Gamma

Superimposed dose profiles of ‘h’ pattern (along the direction of leaf), shown in Figures 7a and 7b, indicate that the measured spatial dose distribution is in agreement with TPS-calculated values. This ensures the validity of the leaf transmission and dosimetric leaf separation value estimated and fed to the TPS.

### B. Dosimetric tests

1. Stability of dMLC output: Measured outputs for sweeping dMLC at all gantry angles showed that the variation was within acceptable limits [Table 3]. This ensures that there is no influence of gravity on the delivered output for 1 cm sweeping gap.

**Table 3:** Variation in output at different Gantry Angles

*SI. No.*	*Gantry angle*	*Off axis (cm)*	*Normalized to 10×10 cm^2^ open field*
1	0	x= 0, z= 0	0.1448
		x= −2.5	0.1448
		x= +2.5	0.1440
		y= +2.5	0.1451
		y= −2.5	0.1456
2	90	x=0, z=0	0.1443
		y= 2.5	0.1446
		y= −2.5	0.1455
		z= +2.5	0.1439
		z= −2.5	0.1443
3	270	x=0, z=0	0.1446
		y= 2.5	0.1453
		y= −2.5	0.1457
		z= +2.5	0.1453
		z= −2.5	0.1444

**Figure 8:**
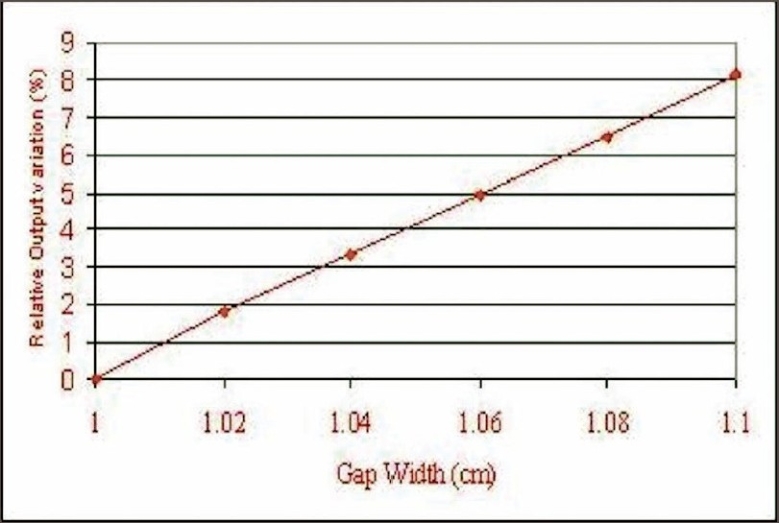
Output variation for sweeping gap for different gap widths

**Figure 9:**
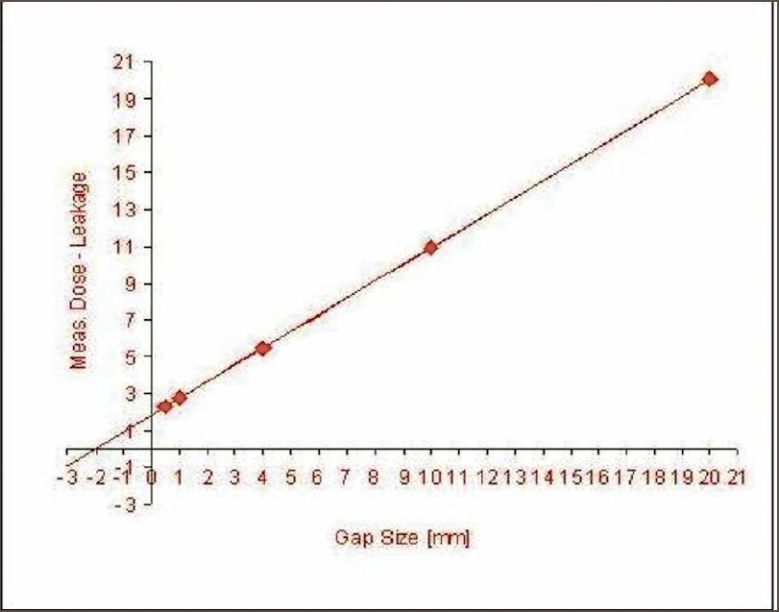
Dosimetric leaf separation determined by sweeping gap technique. Extrapolation of corrected chamber reading versus leaf gap to zero chamber reading gives the DLS

**Figure 10:**
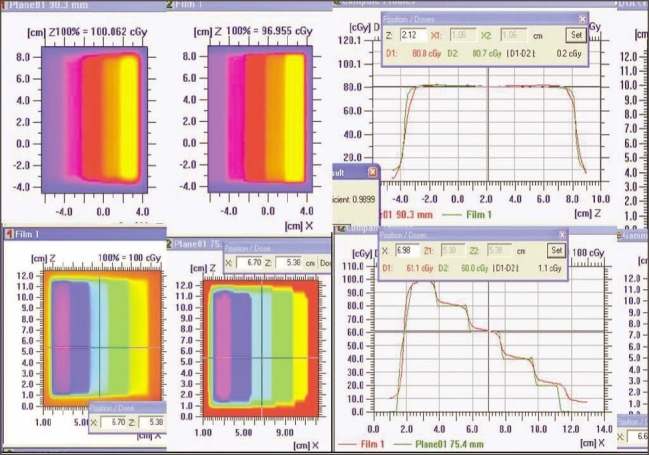
Varying density pattern of X wedge (left top and bottom) and superimposed dose profile parallel to the direction of leaf movement (top right) and quantitative evaluation of superimposed dose profile with DTA (distance to agreement)

2. Variation in output for different sweeping gap widths: Our measurements showed a mean change in output of machine not exceeding 1.6% for a change of 0.02 cm gap width. The variation in the relative output of the sweeping gap versus gap width is seen in Figure 8.

3. Dosimetric leaf gap separation: The dosimetric leaf separation for 6-MV photon determined by sweeping gap technique was found to be 1.9 mm [Figure 9].

4. Dosimetric verification of IMRT delivery and computing system: For different test patterns generated, the doses computed by the TPS and those recorded on the film were well within acceptable limits (both by qualitative and quantitative comparisons). The dose profile pattern (parallel and perpendicular to direction of leaf movement) for the X wedge calculated by TPS and measured on the film [Figure 10] demonstrated good agreement.

5. Validation of IMRT dose calculation and Monitor units: Monitor units, fluence, and dose at reference points calculated by ‘Diamond’ software match well (majority within ±3% and rarely ±5%) with TPS values. The parameters (monitor units, dose at reference point, and the fluence pattern) calculated by ‘Diamond’ software for X wedge pattern, pyramid shape pattern, and for a patient plan are shown in Figures 11a, 11b, 12a, and 12b. These parameters are in good agreement with those planned by TPS.

**Figure 11a:**
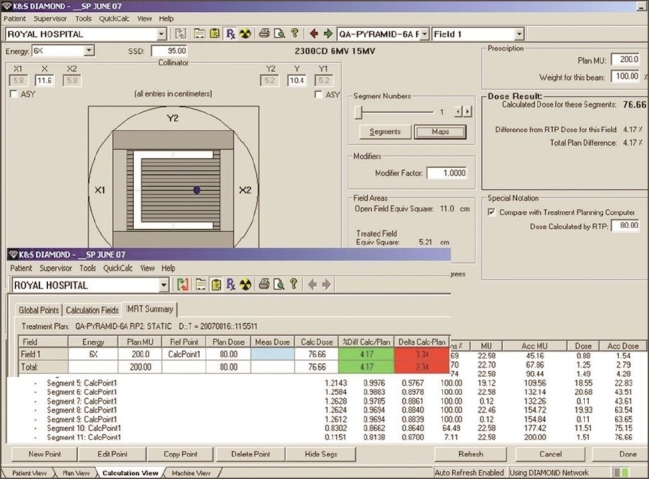
Diamond software showing plan and calculation details of pyramid pattern

**Figure 11b:**
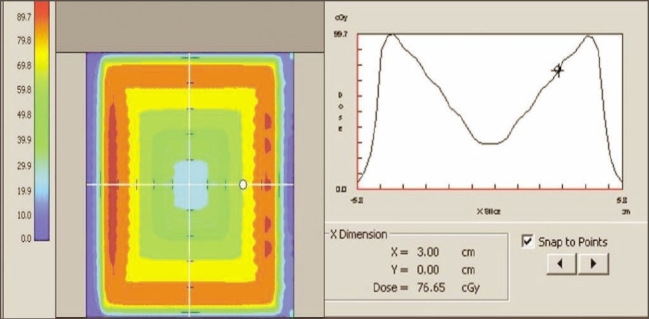
Dose profile calculated by Diamond software indicating the dose at reference point for a IMRT plan

**Figure 12a:**
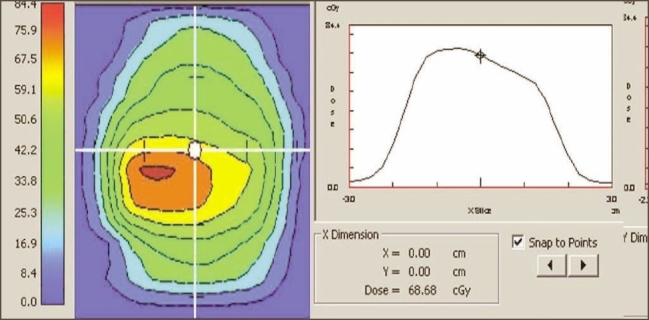
Dose profile calculated by Diamond software indicating the dose at reference point for a IMRT plan

**Figure 12b:**
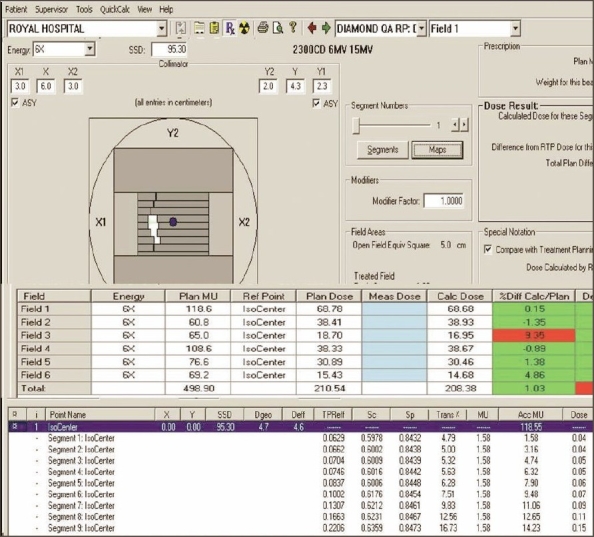
Diamond software showing plan and calculation details of IMRT plan

## Discussion

Sculpting the dose distribution closely to the shape of the tumor and thereby minimizing the dose to the adjacent critical organs with potential for dose escalation to the tumor is the salient feature of IMRT. However, leaf positioning inaccuracies as low as sub-millimeter levels even may lead to erroneous dose delivery. Visual inspection of the recorded density pattern in QA tests can detect misalignment in the leaf position. Qualitative evaluation of standard dMLC test pattern as part of precommissioning test for IMRT implementation is adequate.[6] In addition to qualitative verification of test patterns, we performed quantitative comparison of measured and calculated dose distribution for dMLC patterns (X wedge, Chess shape and h shape) using evaluation tools such as Gamma and distance to agreement (DTA) of Omnipro IMRT software. Quantitative analysis of studied patterns confirmed the accuracy of fractional dose delivery when MLC leaf pairs were operated in dynamic mode. The observed dose differences (among the dose profiles recorded at four different gantry angles) in the Garden Fence intensity patterns are likely to be due to error in film placement on shielding tray holder and the uncertainty associated with the film dosimetry. The variation seen in dose profiles of the film and TPS calculations may be attributed to the scatter radiation and uncertainty associated with the film dosimetry. Within the limits of above inaccuracies, our test patterns were well acceptable.

The influence of leaf speed and stability was evaluated with leaf speed test. If treatment is terminated prematurely, resuming it at the exact point where the original treatment was interrupted is necessary. If acceleration and deceleration effects of the leaves are significant, it will result in observable deviations from the original dose profiles while the treatment is interrupted and restarted. Our test results confirmed that such changes do not occur, revealing that the patient dose remains the same.

While positional inaccuracy of leaf pairs affects the dose at the field boundary in 3D-CRT, in IMRT it affects the dose along the entire swept length of each leaf. Measured output variation of 1.6% for 0.2 mm error in the gap width highlights the need for crucial leaf positional accuracies.

Due to rounded shape of the leaf at their edges, certain amount of radiation passes between the leaves even when a leaf pair is completely closed (rounded leaf edge transmission). DLS accounts for dose transmission through the rounded MLC leaves and additional dose to patient during dynamic dose delivery. The measured DLS value (1.9 mm) is in agreement with the reported values, ranging from 1.9 mm to 2.6 mm.[10] TPS-calculated distribution and measured dose distribution for ‘h’-shaped test pattern were comparable and quantified using evaluation tools such as Gamma and DTA. This test ensures that measured and TPS-fed leaf configuration parameters such as average leaf transmission and dosimetric leaf separation are correct.

IMRT QA remains a challenging and complex task for physicists. IMRT plan validation can be classified into two categories, namely, experimental measurement and independent calculation. Manual verification of dose at a point (or MUs) calculated by the TPS for multiple beam arrangement for IMRT plans remains complex and has limitations. The accuracy of calculation of dose and MUs by Eclipse TPS was verified by the commercial software ‘Diamond’ and reported in the present work.

Relative and absolute dosimetric measurements for patient-specific pretreatment QA are labor intensive and have their own limitations depending on the dosimetry systems used. Positioning errors in ion chamber placement and role of chamber volume in dose gradient regions lead to inaccurate estimations of absolute dose in IMRT. A number of reports have suggested verification of point dose (or MUs) using independent software as a tool for IMRT plan validation.[11,12] Eclipse calculates the final dose distribution and monitor units based on pencil beam algorithm. The leaf motion calculator software of Eclipse converts the optimal fluence into actual fluence, taking into account MLC characteristics and their mechanical constraints. Dose calculations by ‘Diamond’ software is based on modified Clarkson calculations with a ‘points - eye - view’ of head scatter, taking into account the MLC leaf characteristics such as interleaf, intra-leaf, and leaf end transmission. Independent verification of point dose (or MUs) by ‘Diamond’ software helps to validate the IMRT plan done by Eclipse.

Recording the garden fence test pattern on EPID saves manual labor and time delay in film processing. Our observation in the past one year showed no deviation in the MLC leaf alignments, indicating consistent functional performance of the millennium MLC. Positional accuracy of millennium MLC is further confirmed by DynaLog File Viewer software, which tabulates positional error of any individual leaf in terms of its magnitude and frequency of repetition during IMRT delivery.

## Conclusion

The advantage of improved dose conformity to the target and sparing of normal tissues by IMRT is widely appreciated by the radiation oncologists, and IMRT has rapidly become the preferred treatment technique in cancer care in the past few years in developing countries. This complex treatment technique relies on the performance of many hardware components of the linac and related software. Systematic commissioning and QA are integral efforts in the implementation of IMRT. Implementation of IMRT should not be underestimated and oversimplified. Further, the reliability of the implemented IMRT system needs to be periodically verified by quality control surveillance protocol.

This work illustrated in detail the importance of measuring various MLC parameters as also of additional quality assurance tests to be performed on the MLC-equipped linacs before starting IMRT treatment in a radiotherapy clinic. Independent dose verification by commercial software may be considered as an alternative option to relative dosimetry and is highly recommended in centers with high IMRT patient throughput. The mechanical and dosimetric stability of linac for dMLC mode of operation was found to be satisfactory and reliable for clinical implementation of IMRT at our center.
